# A multicentre, prospective study of plasma circulating tumour DNA test for detecting *RAS* mutation in patients with metastatic colorectal cancer

**DOI:** 10.1038/s41416-019-0457-y

**Published:** 2019-04-24

**Authors:** Hideaki Bando, Yoshinori Kagawa, Takeshi Kato, Kiwamu Akagi, Tadamichi Denda, Tomohiro Nishina, Yoshito Komatsu, Eiji Oki, Toshihiro Kudo, Hiroshi Kumamoto, Takeharu Yamanaka, Takayuki Yoshino

**Affiliations:** 1 Department of Gastroenterology and Gastrointestinal Oncology, National Cancer Center Hospital East, Kashiwa, Chiba Japan; 2 Department of Surgery, Kansai Rosai Hospital, Amagasaki, Hyogo Japan; 3 Department of Surgery, National Hospital Organization Osaka National Hospital, Osaka, Osaka Japan; 4 Division of Molecular Diagnosis and Cancer Prevention, Saitama Cancer Center, Ina, Saitama, Japan; 5 Division of Gastroenterology, Chiba Cancer Center, Chiba, Chiba Japan; 6 Department of Gastrointestinal Medical Oncology, National Hospital Organization Shikoku Cancer Center, Matsuyama, Ehime Japan; 7 Department of Cancer Chemotherapy, Hokkaido University Hospital Cancer Center, Sapporo, Hokkaido Japan; 8 Department of Surgery and Science, Graduate School of Medical Science, Kyushu University, Fukuoka, Fukuoka Japan; 9 Department of Frontier Science for Cancer and Chemotherapy, Graduate School of Medicine, Osaka University, Suita, Osaka Japan; 10 Scientific Affairs Division, Clinical Affairs, Sysmex Corporation, Kobe, Hyogo Japan; 11 Department of Biostatistics, Yokohama City University School of Medicine, Yokohama, Kanagawa Japan

**Keywords:** Colorectal cancer, Tumour biomarkers, Cancer genomics

## Abstract

**Background:**

OncoBEAM^TM^ RAS CRC kit using BEAMing technology is a circulating tumour DNA (ctDNA) test for detecting plasma *RAS* mutational status in metastatic colorectal cancer (mCRC). We conducted a multicentre, prospective study to investigate the concordance of the *RAS* mutational status between plasma ctDNA and tumour tissue DNA.

**Methods:**

mCRC patients without prior anti-EGFR antibodies or regorafenib treatment were enroled. Plasma- and tissue-based *RAS* mutational status were determined by BEAMing, respectively.

**Results:**

A total of 280 patients from eight institutions were eligible. The overall agreement between plasma- and tissue-based analyses was 86.4%, with a positive percent agreement of 82.1% and negative percent agreement of 90.4%. From logistic regression analysis, lung metastasis alone indicated the most significant factor associated with discordance. The agreement between plasma- and tissue-based analyses was 64.5% in patients with lung metastasis alone (*n* = 31) indicating lower amount of ctDNA. Among the cases with lung metastasis alone, all plasma- and tissue-based analyses were perfectly concordant in cases with ≥20 mm of maximum lesion diameter or ≥10 lesions.

**Conclusion:**

The clinical validity of OncoBEAM^TM^ RAS CRC kit was confirmed. Careful attention should be paid for mCRC patients with lung metastases alone having fewer metastases or smaller diameter lesions.

## Background

Colorectal cancer (CRC) is the third most commonly diagnosed cancer and the fourth most common cause of cancer deaths worldwide. Although the epidermal growth factor receptor (EGFR) has become an important therapeutic target for CRC treatment, approximately 40% of patients with metastatic colorectal cancer (mCRC) have tumours with *KRAS* mutations, which are not expected to respond to anti-EGFR therapies.^1,2^ Furthermore, numerous comprehensive prospective or retrospective analysis of *KRAS* and *NRAS* codons 12, 13, 59, 61, 117, and 146 demonstrated that patients with these mutations did not receive clinical benefits from anti-EGFR therapies.^3^

Cell-free DNA (cfDNA) is the natural DNA present in the cell-free fraction of the blood. Recent studies have suggested that genomic alterations in solid tumours can be characterised by studying the circulating-tumour DNA (ctDNA), which is the cfDNA released from cancer cells into the plasma.^4^ While ctDNA can exist in almost all patients with mCRC, its low abundance requires highly sensitive techniques to detect the mutations presenting at low frequencies. The benefits of investigating the ctDNA are: provisions of a rapid genotype result with a streamlined clinical workflow and minimal patient invasiveness.

The OncoBEAM^TM^ RAS CRC Kit, which uses BEAMing digital PCR technology, is a CE-marked in vitro diagnostic based in Europe used for detecting *RAS* mutations in ctDNA derived from mCRC. Several prospective or retrospective studies to compare the *RAS* mutational status determined by BEAMing in plasma and the tissue reference method have been associated with concordance rates from 89.7% to 93.3%.^5–7^ Recently, García-Foncillas et al. demonstrated a concordance rate of 92.0% (*n* = 236) between the plasma-BEAMing and tissue reference method. The analysis of 8.0% of discordant cases suggested that the higher discordances were observed in the patient with lung metastasis alone.^8^

Herein, we have conducted a prospective multicentre trial to investigate the concordance between the mutational status of *RAS* determined by plasma- and tissue-based BEAMing in patients with mCRC; we have also examined the characteristics of discordant cases.

## Materials and methods

### Study design and patients

This prospective study was carried out in eight Japanese medical centres from June 2017 to February 2018 (UMIN000023039). Patients with pathologically confirmed mCRC who were chemo-naive or confirmed to have progressive disease (PD) without having initiated subsequent treatment and had an adequate archived formalin-fixed paraffin embedded (FFPE) tumour tissue specimen obtained within 5 years were enroled. Patients who had received prior treatment with anti-EGFR antibodies or regorafenib were excluded according to the possible appearance of acquired resistant mutations.^9–11^ This study was approved by the ethics committees at each institution (National Cancer Center Institutional Review Board, Kansai Rosai Hospital Review Board, Saitama Cancer Center Review Board, Chiba Cancer Center Review Board, National Hospital Organization Shikoku Cancer Center Review Board, Hokkaido University Review Board, Kyushu University Review Board, and Osaka University Review Board), and all patients provided written informed consent. All procedures related to the study were performed in accordance with the Helsinki Declaration.

### Procedures

Plasma was obtained from 10 ml of blood collected by Streck Cell-Free DNA BCT® Tube or BD Vacutainer® K2 EDTA Tube. FFPE tissues with >30% tumour area were used for analysis. DNA was isolated from blood and FFPE by QIAamp Circulating Nucleic Acid Kit and QIAamp DNA FFPE, respectively. Genome equivalent (GE) of isolated DNA was quantified by LINE-1 quantitative real-time PCR assay as described previously.^12^

OncoBEAM^TM^ RAS CRC Kit, which detects 34 mutations in *KRAS*/*NRAS* codons 12, 13, 59, 61, 117, and 146, was used to analyse *RAS* mutations in plasma (plasma-BEAMing) at the Sysmex IMP laboratory, using the cut-off defined as the number of beads with amplified-mutant molecules specifically set per each codon (Kobe, Japan). FFPE tissue was used to analyse the 33 in *KRAS*/*NRAS* codons 12, 13, 59, 61 (*KRAS* Q61R is excluded as compared with plasma-BEAMing), 117, and 146 by BEAMing, as a reference method (tissue-BEAMing), using a 5% cut-off at Sysmex Inostics (Hamburg, Germany). MEBGEN^TM^ RASKET Kit (RASKET),^13^ which is approved as a companion diagnostic in Japan, was utilised to detect *RAS* mutations in FFPE tissue to validate the accuracy of tissue-BEAMing. Oncomine^TM^ Colon cfDNA assays^14^ (plasma-NGS) using a cut-off of 0.05% were also utilise for reflex testing of all the cases where the plasma-BEAMing result was discordant with the tissue-BEAMing result. We also randomly sampled 100 cases from all the enroled cases and performed the plasma-NGS analysis for confirming the accuracy of plasma-BEAMing.

### Statistical analysis

The primary analysis was to evaluate concordance between plasma- and tissue-*RAS* mutational status by BEAMing based on overall concordance rate, sensitivity (positive percent agreement), and specificity (negative percent agreement), where the evaluation was done for cases having both plasma- and tissue-testing results. Evaluation of plasma-BEAMing with reference to plasma-NGS, as well as that of tissue-BEAMing with reference to RASKET was also performed.

Factors associated with discordance were calculated using the univariate and multivariate logistic regression models. The multivariate analysis was performed using factors with a *P* < 0.1 by the univariate analysis. Statistical analyses were performed using the R i386 3.4.0 software.

## Results

### Patient characteristics

A total of 350 patients with mCRC were initially enroled, 70 of whom were excluded for primary analysis due to the following reasons (Figure S1): lacking the qualified plasma or tissue availability (*n* = 18), invalid results by tissue-BEAMing (*n* = 29), invalid results by plasma-BEAMing (*n* = 8), and conflicting exclusion criteria (*n* = 15). The remaining 280 patients were evaluated as a primary analysis set (Table 1). Most of the patients (67%, 189/280) had recurrent diseases and more than half of the patients (54%, 151/280) were chemo naive at the enrolment. Almost half of the patients (49%, 138/280) had multiple sites of metastatic disease. In patients with only one metastatic site, the most frequent site was the liver (27%, 76/280), followed by the lung (11%, 31/280).

**Table 1:** Patient characteristics

Characteristics		Primary analysis set (*N* = 280)	%
Age	Median, years [range]	67 [61–73]	—
Gender	Male	164	59
Newly diagnosed or relapsed	Newly diagnosis	91	33
Chemo naive	Yes	151	54
Location of primary tumour^a^	Right-sided	91	33
Metastatic site	Liver alone	76	27
	Lung alone	31	11
	Lymph node alone	23	8
	Other single organ	12	4
	Multiple sites	138	49
Sample collection interval from archived tissue to plasma	Median, mo [range]	11 [1–61]	—
Source of tissue samples for test	Primary/metastasis	268/12	96/4

*Chemo* Chemotherapy

^a^Right-sided colon includes transverse colon, ascending colon and cecum and left-sided colon includes descending colon, sigmoid colon and rectum

### Concordance of *RAS* mutational status between plasma and tissue

Overall, *RAS* mutations were detected in 44.3% of plasma samples and in 47.9% of tissue samples. The overall concordance between plasma- and tissue-based analyses was 86.4% (242/280), with a positive percent agreement of 82.1% (110/134) and negative percent agreement of 90.4% (132/146) (Table 2). Of the 38 discordant cases, we tested the 32 cases by plasma-NGS to investigate the reasons of discordances using different plasma assays, except for 6 cases who do not have enough samples for plasma-NGS. Patients with positive plasma-BEAMing and negative tissue-BEAMing results were observed in 14 cases, of which 6 were determined to be positive by plasma-NGS (table S1). On the other hand, patients with negative plasma-BEAMing and positive tissue-BEAMing results were observed in 24 cases, of which 15 were confirmed to be negative by plasma-NGS (table S1). The concordance of the two different plasma methods was 96.0% (Table 3). Similarly, the concordance between tissue-BEAMing and RASKET using the same tissue samples was 93.9% (Table 3).

**Table 2:** Concordance between plasma and tissue-BEAMing

		Tissue-BEAMing		Total	Concordance% [95% CI]	Sensitivity% [95% CI]	Specificity% [95% CI]
		MT	WT				
Plasma-BEAMing	MT	110	14	124	86.4 [81.9–90.2]	82.1 [74.5–88.2]	90.4 [84.4–94.7]
	WT	24	132	156			
Total		134	146	280			

*MT* Mutant, *WT* Wildtype

**Table 3:** Analytical accuracy of BEAMing; Plasma vs Plasma and Tissue vs Tissue

		Tissue-RASKET		Total	Concordance% [95% CI]
		MT	WT		
Tissue-BEAMing	MT	137	11	148	93.9 [90.5–96.3]
	WT	7	139	146	
Total		144	150	294	

		Plasma-NGS		Total	Concordance% [95% CI]
		MT	WT		
Plasma-BEAMing	MT	48	3	51	96.0 [90.1–98.9]
	WT	1	48	49	
Total		49	51	100	

*MT* Mutant, *WT* Wildtype

### Variables associated with discordance

We investigated the logistic regression analysis for identifying the factors associated with the discordance (Table 4). The most significant factor associated with discordance was lung metastasis alone, followed by sample collection interval from archived tissue to plasma. The multivariate analysis revealed that lung metastasis alone independently had a significant association with discordance.

**Table 4:** Comparison of baseline characteristics between concordant and discordant cases

		Odds ratio (Univariate)	*P* value	Odds ratio (Multivariate)	*P* value
Age	—	0.99	0.46	—	—
Gender	Male	0.91	0.79	—	—
Newly diagnosed or relapsed	Relapsed	0.51	0.11	—	—
Chemo naive	Yes	1.2	0.6	—	—
Location of primary tumour	Right-sided	1.26	0.58	—	—
Metastatic site	Liver alone	1.77	0.2	—	—
	Lung alone	0.22	<0.01	0.24	<0.01
	Lymph Node (LN) alone	1.71	0.19	—	—
	Other single organ meta	0.78	0.48	—	—
	Multiple metastasis	1.4	0.74	—	—
Sample collection interval from archived tissue to plasma	—	0.98	0.09	0.99	0.23
Source of tissue sample for test	Primary	2.22	0.25	—	—

Among the patients with lung metastasis alone, the overall agreement between the plasma- and tissue-based analyses was only 64.5% (20/31), while the overall concordance rate was 89.2% (222/249) in case of the patients excluding lung metastasis alone. Notably, the sensitivity of patients with lung metastasis alone was significantly lower than the cases excluding lung metastasis alone (41.2%: 7/17 vs. 88.0%: 103/117) (Table 5).

**Table 5:** Concordance rate in cases with lung metastases alone or with excluding lung metastasis alone

Lung met. alone		Tissue-BEAMing		Total	Concordance% [95% CI]	Sensitivity% [95% CI]	Specificity% [95% CI]
		MT	WT				
Plasma-BEAMing	MT	7	1	8	64.5 [45.4–80.8]	41.2 [18.4–67.1]	92.9 [66.1–99.8]
	WT	10	13	23			
Total	17	14	31				

Excluding lung met. alone		Tissue-BEAMing		Total	Concordance% [95% CI]	Sensitivity% [95% CI]	Specificity% [95% CI]
		MT	WT				
Plasma-BEAMing	MT	103	13	116	89.2 [84.6–92.7]	88.0 [80.7–93.3]	90.2 [83.7–94.7]
	WT	14	119	133			
Total	117	132	249				

*MT* Mutant, *WT* Wildtype

### Analysis of mutation allele frequency (MAF) and lesion volume

**Fig. 1:**
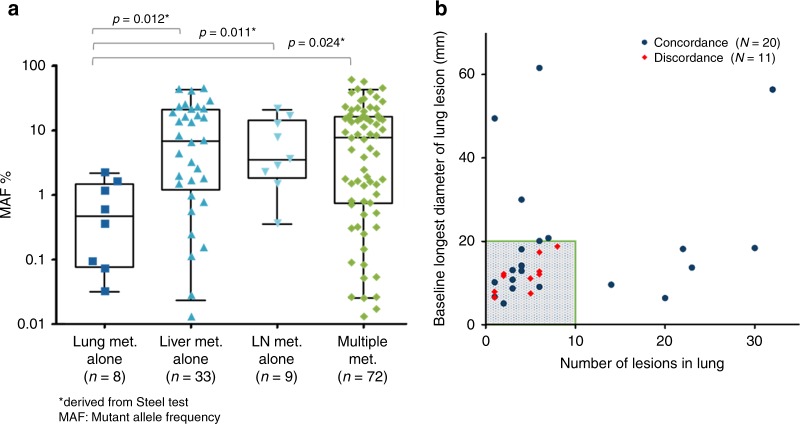
Analysis of mutation allele frequency and lesion volume. Mutation allele frequency (MAF) in cases with metastatic site (**a**) and the longest diameter of lesion and number of lesions with concordant and discordant cases (**b**). *Derived from Steel test with control of group of lung metastasis alone

A clear correlation between the metastatic site and mutation allele frequency (MAF) was observed. The median MAF [range] of the patients who had lung metastasis alone was 0.47% [0.03%–2.21%], which is significantly lower than patients who had other metastatic sites (Overall: 4.75% [0.01–59.76], Liver metastasis alone: 6.92% [0.01%–44.09%], LN metastasis alone: 3.56% [0.36%–21.21%], Multiple metastasis: 8.19% [0.01%–59.76%]) (Fig. 1a). In order to find the trends of lower concordance of patients with lung metastasis alone (*n* = 31), we performed the post-hoc analysis, investigating the diameter and number of metastatic lesions. As shown in Fig. 1b, all discordant cases had the baseline longest diameter of lung lesion of smaller than 20 mm and less than 10 lesions (9/20, 11 cases were discordant.). Conversely, all cases were concordant with the baseline longest diameter of lung lesion equal to or larger than 20 mm, or lesions equal to or more than 10 (11/11) (Fig. 1b).

## Discussion

This is the second clinical study to prospectively investigate the concordance of *RAS* mutational status between plasma- and tissue-BEAMing and the first in Asian patients with mCRC. The concordance rate between plasma- and tissue-BEAMing was 86.4%, which was comparable to those in previous studies (89.7%–93.3%).^5–7^ Of 38 discordant cases, 6 of 14 cases with positive plasma-BEAMing results and negative tissue-BEAMing results were plasma-NGS-positive (table S1). On the other hand, 15 of 24 cases with negative plasma-BEAMing and positive tissue-BEAMing results were plasma-NGS-negative (table S1). These results suggested that over half of the discordant cases (21/38) determined by plasma-BEAMing might be analytically accurate. In addition, a concordance rate of 96.0% between plasma-BEAMing and plasma-NGS was obtained, indicating an almost perfect agreement.

The plausible reason of discordance could be lower ctDNA shedding from tumours. The relevance between MAF burden and concordance were found as that median MAFs [range] with concordant cases and discordant cases were 7.33% [0.03%-59.76%] and 0.04% [0.01%-30.25%] (*p* = 0.001132, wilcoxon rank sum test), respectively. Furthermore, in the logistic regression analysis, lung metastasis alone was the most significant factor associated with discordance, showing the lowest MAF (0.47%), which is consistent with the finding reported by Vidal et al. describing lower MAF in patients with only lung metastatic involvement.^15^ The concordance rate of the patients with lung metastasis alone was down to 64.5%; especially, the sensitivity decreased to 41.2% (7/17), while the specificity was maintained at 92.9% (13/14). These results suggest that the plasma-BEAMing analyses for the cases with lung metastasis alone have a greater likelihood to show false negative results. Garcia-Foncillas et al. also reported that a low concordance rate was associated with the presence of lung metastasis alone.^8^ However, the population of those cases was considerably lower than that in our study [6.8% (16/236) vs. 11.1% (31/280)], which was possibly associated with the slightly lower concordance rate in our study (86.4%), compared to previous studies (89.7%–93.3%).^5–7^ Notably, we performed the post-hoc analysis of cases with lung metastasis alone and identified that the maximum lesion diameter and number of lesions had an impact on the discordant results. Although a limited number of cases had been analysed, the longest diameter of lung lesion of 20 mm, and lesions of 10 are specious cut-offs to discriminate the discordant cases. To furtherly investigate the optimal cut-off value of maximum diameter of lesion and number of lesions to predict concordant results in cases with lung metastasis alone or peritoneal metastasis alone, we are planning meta-analysis by combining our data with those in the previous western studies.

Other reasons for discordance could be clonal evolution and/or tumour heterogeneity. In the univariate logistic regression model, we found trends of the association of sample collection interval with discordance, suggesting clonal evolution due to longer intervals between plasma and tissue collections. Cases with heterogeneous tumours might also be included in plasma-positive and tissue-negative populations. Further investigation such as multiple-lesions or -times from tissue samples, and longitudinal assessment by plasma-NGS assessment to examine the mutational status of various genes, may lead the clarification of the tumour heterogeneity and clonal evolution. Additionally, investigation of efficacy with anti-EGFR antibodies in discordant cases, especially case with longer intervals between plasma and tissue collections, will establish the real value of OncoBEAM^TM^ RAS CRC Kit.

In conclusion, the reliability of plasma *RAS* mutational status determined by OncoBEAM^TM^ RAS CRC Kit was confirmed in Asian patients with mCRC. Careful attention should be paid to interpret the results when we use the plasma-BEAMing for mCRC patients with lung metastases alone having fewer lung metastases as well as those with smaller diameter lesions.

## Supplementary information

Supplementary files

The name of all ethic committee

## Data Availability

All data generated or analysed during this study are included in this published article and its supplementary information files.
