# Correction: A multicentre, prospective study of plasma circulating tumour DNA test for detecting *RAS* mutation in patients with metastatic colorectal cancer

**DOI:** 10.1038/s41416-020-0766-1

**Published:** 2020-02-25

**Authors:** Hideaki Bando, Yoshinori Kagawa, Takeshi Kato, Kiwamu Akagi, Tadamichi Denda, Tomohiro Nishina, Yoshito Komatsu, Eiji Oki, Toshihiro Kudo, Hiroshi Kumamoto, Takeharu Yamanaka, Takayuki Yoshino

**Affiliations:** 1 Department of Gastroenterology and Gastrointestinal Oncology, National Cancer Center Hospital East, Kashiwa, Chiba Japan; 2 Department of Surgery, Kansai Rosai Hospital, Amagasaki, Hyogo Japan; 3 Department of Surgery, National Hospital Organization Osaka National Hospital, Osaka, Osaka Japan; 4 Division of Molecular Diagnosis and Cancer Prevention, Saitama Cancer Center, Ina, Saitama Japan; 5 Division of Gastroenterology, Chiba Cancer Center, Chiba, Chiba, Japan; 6 Department of Gastrointestinal Medical Oncology, National Hospital Organization Shikoku Cancer Center, Matsuyama, Ehime Japan; 7 Department of Cancer Chemotherapy, Hokkaido University Hospital Cancer Center, Sapporo, Hokkaido Japan; 8 Department of Surgery and Science, Graduate School of Medical Science, Kyushu University, Fukuoka, Fukuoka Japan; 9 Department of Frontier Science for Cancer and Chemotherapy, Graduate School of Medicine, Osaka University, Suita, Osaka Japan; 10 Scientific Affairs Division, Clinical Affairs, Sysmex Corporation, Kobe, Hyogo Japan; 11 Department of Biostatistics, Yokohama City University School of Medicine, Yokohama, Kanagawa Japan

**Keywords:** Colorectal cancer, Tumour biomarkers, Cancer genomics

**Correction to**: *British Journal of Cancer* 10.1038/s41416-019-0457-y, published online 24 April 2019

This Article was originally published under Nature Research’s License to Publish, but has now been made available under a CC BY 4.0 license. The PDF and HTML versions of the Article have been modified accordingly.

