# Supplementary material for: A multicentre, prospective study of plasma circulating tumour DNA test for detecting RAS mutation in patients with metastatic colorectal cancer
Source: Br J Cancer. 2019 Apr 24;120(10):982–6. doi: 10.1038/s41416-019-0457-y (PMC6734650; doi:10.1038/s41416-019-0457-y)

Table S1 Characteristics with discordant cases.


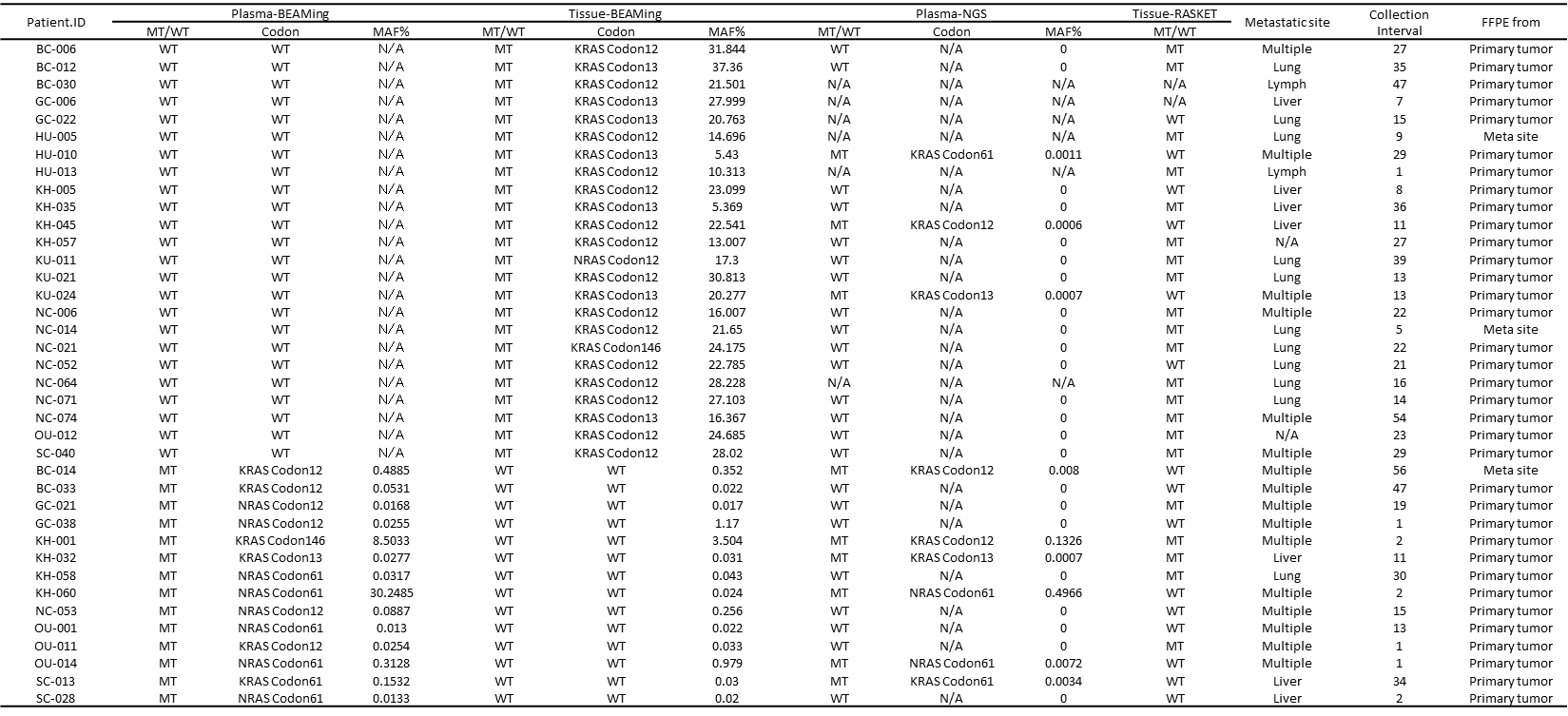


MT: Mutant, WT: Wildtype, MAF: Mutant allele frequency, N/A: Not available, Collection interval: time length from tissue sampling to plasma sampling.


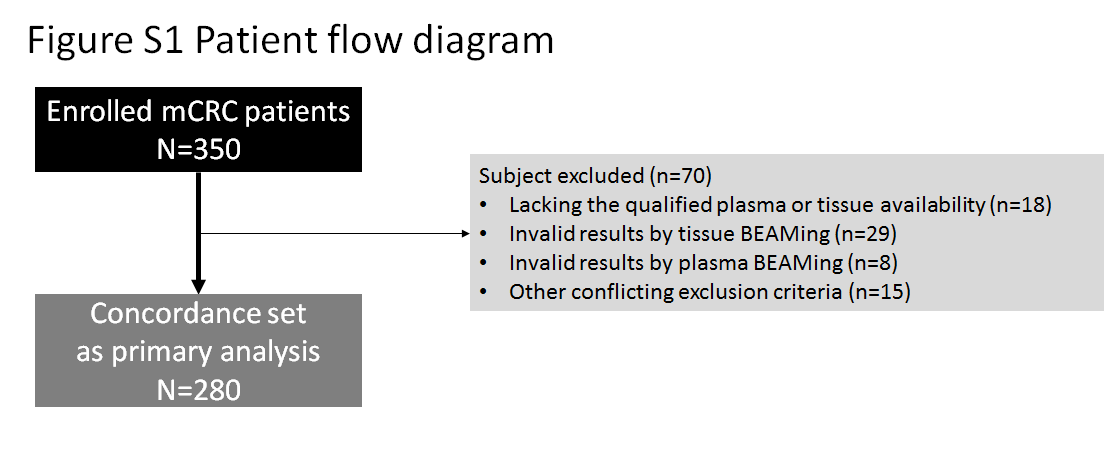

Supplement: Supplementary file 1 — Supplementary files [file 41416_2019_457_MOESM1_ESM.docx]
