# Supplementary material for: A multicentre, prospective study of plasma circulating tumour DNA test for detecting RAS mutation in patients with metastatic colorectal cancer
Source: Br J Cancer. 2019 Apr 24;120(10):982–6. doi: 10.1038/s41416-019-0457-y (PMC6734650; doi:10.1038/s41416-019-0457-y)
Supplement: Supplementary file 2 — The name of all ethic committee [file 41416_2019_457_MOESM2_ESM.docx]

The name of all ethic committees

National Cancer Center Institutional Review Board,

Kansai Rosai Hospital Review Board,

Saitama Cancer Center Review Board,

Chiba Cancer Center Review Board,

National Hospital Organization Shikoku Cancer Center Review Board,

Hokkaido University Review Board,

Kyushu University Review Board

Osaka University Review Board
